# Occupational Traumatic Injuries Among Workers in Health Care Facilities — United States, 2012–2014

**Published:** 2015-04-24

**Authors:** Ahmed E. Gomaa, Loren C. Tapp, Sara E. Luckhaupt, Kelly Vanoli, Raymond Francis Sarmiento, William M. Raudabaugh, Susan Nowlin, Susan M. Sprigg

**Affiliations:** 1 Division of Surveillance, Hazard Evaluations and Field Studies, National Institute for Occupational Safety and Health; 2 Public Health Informatics Fellowship Program, Division of Scientific Education and Professional Development, Center for Surveillance, Epidemiology and Laboratory Services, CDC

In 2013, one in five reported nonfatal occupational injuries occurred among workers in the health care and social assistance industry, the highest number of such injuries reported for all private industries (1). In 2011, U.S. health care personnel experienced seven times the national rate of musculoskeletal disorders compared with all other private sector workers (2). To reduce the number of preventable injuries among health care personnel, CDC’s National Institute for Occupational Safety and Health (NIOSH), with collaborating partners, created the Occupational Health Safety Network (OHSN) to collect detailed injury data to help target prevention efforts. OHSN, a free, voluntary surveillance system for health care facilities, enables prompt and secure tracking of occupational injuries by type, occupation, location, and risk factors. This report describes OHSN and reports on current findings for three types of injuries. A total of 112 U.S. facilities reported 10,680 OSHA-recordable* patient handling and movement (4,674 injuries); slips, trips, and falls (3,972 injuries); and workplace violence (2,034 injuries) injuries occurring from January 1, 2012–September 30, 2014. Incidence rates for patient handling; slips, trips, and falls; and workplace violence were 11.3, 9.6, and 4.9 incidents per 10,000 worker-months,† respectively. Nurse assistants and nurses had the highest injury rates of all occupations examined. Focused interventions could mitigate some injuries. Data analyzed through OHSN identify where resources, such as lifting equipment and training, can be directed to potentially reduce patient handling injuries. Using OHSN can guide institutional and national interventions to protect health care personnel from common, disabling, preventable injuries.

OHSN is a web-based data portal that accepts health care facilities’ existing OSHA-recordable and non-recordable health care personnel injury data. De-identified injury data are converted to standard OHSN data elements designed to characterize first, the occupation of the injured worker; second, the type, severity, cause and location of the injury; and finally, information useful in determining how the injury could be prevented. Standardization of data across all facilities allows comparison within and across facilities; comparison groups can be selected by OHSN participants (e.g., hospitals of comparable size or in the same geographic region). New data submissions are available to OHSN participants within a week, and they can analyze new and historical injury data and produce outputs in the form of graphs and tables at any time. The NIOSH OHSN topic page provides information on 1) data terminology, transmission, and security; 2) examples of output graphs and tables; and 3) intervention resources (3).

OHSN received data on injuries occurring from January 1, 2012–September 30, 2014, from 112 U.S. health care facilities. Pooled mean incidence rates§ and percentiles were calculated for three types of OSHA-recordable injuries: 1) falls, including slipping or tripping without a fall; 2) patient handling (e.g., handling, pushing, pulling, or lifting patients); and 3) workplace violence (i.e., violent acts directed at health care personnel). For each of the three injury types, the same denominator was used for all sub-analyses within an injury type, because more specific denominators were not available.

The 112 participating facilities were located in 19 states, with 52% located in the Midwest. By size, 46% had bed numbers of less than 200 and by type, 95% were general medical and surgical facilities. The participating facilities had a total of 162,535 full-time employees and reported a total of 13,798 slips, trips, and falls; patient handling; and workplace violence injuries; of this total, 10,680 (77.4%) were OSHA-recordable injuries. Overall incidence rates of OSHA-recordable injuries (average worker-months = 125,041) per 10,000 worker-months for patient handling; slips, trips and falls; and workplace violence were 11.3, 9.6, and 4.9, respectively (Table). Most injuries occurred in two groups of workers, those aged 30–44 years (35%) and those aged 45–64 years (44%). Nurses (38%) and nursing assistants (19%) accounted for 57% of identified OSHA-recordable injuries. Between 70%–90% of OSHA-recordable patient handling; slips, trips, and falls; and workplace violence injuries occurred among female employees.

Nurse assistants were more likely to sustain injuries than workers in other job categories; this occupation had more than twice the injury rate of nurses for patient handling and workplace violence injuries (Figure 1). Injury rates for slips, trips, and falls were highest among nonpatient care staff (e.g., maintenance and security staff), nursing assistants, and nurses. Between 2012 and 2014, workplace violence injury rates increased for all job classifications and nearly doubled for nurse assistants and nurses (Figure 2). Patient handling and workplace violence injury rates were highest in inpatient adult wards; these rates were also elevated in outpatient emergency departments, urgent care, and acute care centers and adult critical care departments. Rates of falls were highest in inpatient adult wards, nonpatient care maintenance areas, and operating rooms (Table).

Of all patient handling injury reports, 62% included data on the use of lifting equipment; 82% of the injuries occurred when lifting equipment was not used (Table). Of all slips, trips and falls injury reports, 65% had data on fall type; 89% were falls on the same level, 9% were falls to a lower level (e.g., down stairs, ramps, etc.) and 2% were slips and trips without falling. Of all workplace violence injury reports, 49% specified type of assault (physical, verbal, or destruction of property); 99% were physical assaults. Descriptions of who perpetrated the assaults were included in 13% of workplace violence injury reports; 95% were committed by patients which is in agreement with previous study findings (4).

## Discussion

This report examines patient handling; slips, trips, and falls; and workplace violence injuries, which make up a substantial portion of all occupational injuries in the health care sector, as reported by the national Bureau of Labor Statistics findings for workers in all sectors (5). Overall, for the 112 OHSN participating facilities, rates of patient handling and workplace violence injuries were highest among nurse assistants and nurses; rates of slips, trips, and falls were high for these jobs and also for nonpatient care staff. In contrast, physicians, dentists, interns, and residents have low injury rates. These data indicate that interventions should first focus on prevention of injuries to nurse assistants and nurses from patient handling; slips, trips, and falls; and workplace violence. Patient handling and workplace violence injuries reported to OHSN were clustered in locations providing direct patient care, while slips, trips, and fall injuries occurred in both patient and non-patient areas. Analysis of detailed, facility-level data could identify the higher risk occupations and locations of each facility and assist in customizing prevention measures.

Other studies found that musculoskeletal disorders are increasing among health care personnel (2). Nursing staff are exposed to several musculoskeletal disorder risk factors: 1) caring for overweight/obese and acutely ill patients; 2) high patient-to-nurse ratios; 3) long shifts; and 4) current efforts to mobilize patients almost immediately after medical interventions (6). Prevention measures might concentrate on mitigating the high-risk aspects of these jobs. Similar to findings from other studies, OHSN data indicate that interventions (e.g., the use of lifting equipment) could potentially reduce patient-handling injuries, particularly for activities involving positioning, transferring, or lifting a patient (7). Additionally, to prevent patient-handling injuries, health care institutions might establish a safety culture emphasizing continuous improvement and also provide resources such as training in safe patient handling and access to lifting teams and lifting equipment. On the basis of OHSN findings, the major causes of slip, trip, and fall injuries are floor contaminants and contact with objects; however, the variability in types of these injuries indicates that each facility should use facility-specific data to guide prevention measures. The OHSN topic page provides links to helpful resources on safe patient handling methods and prevention of falls among health care personnel, including a comprehensive falls hazards checklist (3).

In 2013, Bureau of Labor Statistics found rates of injuries and illnesses resulting from workplace violence increased for the second year in a row to 16.2 cases per 10,000 full-time workers in the health care and social assistance sector (5). Data reported to OHSN revealed the same trend. The OHSN topic page provides links to workplace violence prevention resources, including an online course to help hospital staff with identifying patients at risk for committing violent acts (those with mental illness, behavioral disorders, and cognitive dysfunction) as well as ways to moderate and prevent violent patient behavior (3).

The findings in this report are subject to at least four limitations. First, in 2012–2014, only 112 U.S. health care facilities from 19 states participated, and the data in this report might not be very representative of the thousands of health care facilities in the United States. Second, a considerable proportion of OHSN injury data regarding risk factors are categorized as unspecified, which could limit OHSN’s ability to identify causality and prevention needs. Third, possible participation, reporting, and recording biases might exist. Voluntary participation might skew participation to best-practice facilities and some facilities might not report all injury data, leading to underestimation of injury rates. Not all facilities collect detailed data requested by OHSN, such as specific activities which lead to patient-handling injuries or why a patient or coworker commits violence against health care personnel. Thus, missing data might bias the results. As participating facilities submit more complete information on worker injuries, the large amount of unspecified data might likely diminish. NIOSH personnel can assist facilities with improving data completeness and quality.

OHSN offers a variety of tools for NIOSH and health care institutions to work toward a common goal of employee safety and health by reducing all types of injuries among health care personnel. OHSN enables health care facilities to track injuries; collect and analyze detailed standard injury data to direct resources toward employees, departments, and situations most at risk; compare their own injury rates with groups of their choosing; access prevention resources; facilitate implementation of timely prevention measures; and monitor intervention impact. Emphasizing worker safety promotes and strengthens patient safety (8), which contributes to improved patient care and reduced costs (9). Future improvements to OHSN include plans to develop a module to systematically collect detailed information on occupational injuries from needles, scalpels, and other sharp objects, and blood and body fluid exposures among health care personnel to assist in creating prevention strategies for those hazards. Targeting prevention strategies can protect health care personnel from prevalent, disabling injuries and help in managing resources.

What is already known on this topic?

The health care and social assistance sector accounts for the greatest proportion (20.7%) of private industry nonfatal occupational injuries among all sectors. The most common injuries are due to patient handling; slips, trips, and falls; and workplace violence.

What is added by this report?

The Occupational Health Safety Network (OHSN) collects and reports near real-time, specific, standard benchmarking information on injuries to help target prevention measures toward workers, departments, and activities at highest risk. From January 1, 2012 to September 30, 2014, the highest incidence rates of the three categories of occupational injuries were among nurse assistants and nurses. Workplace violence injury incidence rates increased from 2012 to 2014; most of these injuries were physical in nature and caused by patients. In over half of patient handling injuries, lifting equipment was not used (51%).

What are the implications for public health practice?

Injury prevention interventions mitigating high-risk aspects of nurse and nurse assistant duties are needed. Safety cultures that emphasize continuous improvement and support resources such as routine use of lifting equipment, as well as safe patient-handling training and lifting teams, might prevent many of the musculoskeletal disorders from patient handling and the associated costs of diagnosis, treatment, and disability.

## Figures and Tables

**FIGURE 1:**
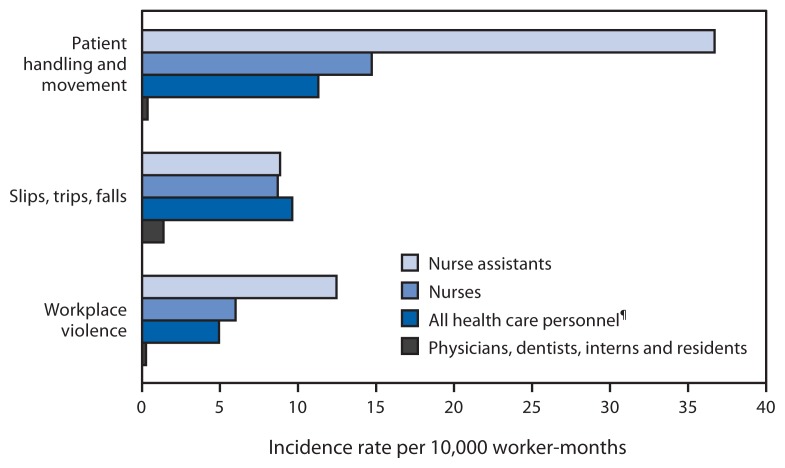
Comparison of OSHA-recordable* injury incidence rates^†^ per 10,000 worker-months^§^ by occupation groups among 112 U.S. health care facilities, January 1, 2012–September 30, 2014 **Abbreviations:** OSHA = Occupational Safety and Health Administration. *OSHA-recordable injuries are defined as work-related injuries and illnesses that result in at least one of the following: death, loss of consciousness, days away from work, restricted work activity or job transfer, medical treatment beyond first aid, or a diagnosis by a physician or other licensed health care professional. ^†^ Injury incidence rate = (number of injuries/total facility full-time employees) × 10,000. ^§^ Worker-months are the number of full-time equivalent workers at a facility (or group of facilities) multiplied by the number of months worked within the reporting period. For example, a facility with 1,000 full-time equivalent workers has 12,000 worker-months in a 12 month reporting period. Worker-months are specific for each occupation (e.g., only full-time equivalent nurses are used to calculate incidence rates for nurses). ^¶^ Nonpatient care staff is included in all health care personnel.

**FIGURE 2:**
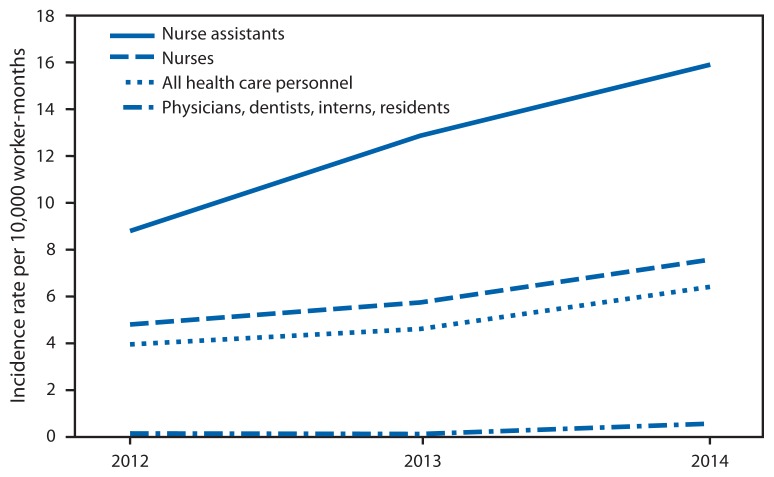
Comparison of OSHA-recordable workplace violence injury incidence rates per 10,000 worker-months* by year among 112 U.S. health care facilities, January 1, 2012–September 30, 2014 **Abbreviation:** OSHA = Occupational Health and Safety Administration. * Worker-months are the number of full-time equivalent workers at a facility (or group of facilities) multiplied by the number of months worked within the reporting period. For example, a facility with 1,000 full-time equivalent workers has 12,000 worker-months in a 12-month reporting period. Worker-months are specific for each occupation (e.g., only full-time equivalent nurses are used to calculate incidence rates for nurses).

**TABLE:** Incidence rates* of OSHA-recordable† slips, trips, and falls; patient handling and movement; and workplace violence injuries per 10,000 worker-months§ by selected categories — Occupational Health Safety Network (OHSN), 112 U.S. health care facilities (HCFs) January 1, 2012–September 30, 2014

Category	No. of reporting HCFs	No. of injuries	Pooled mean incidence rate¶	Incidence rate percentiles		

				25%	50%	75%
**Patient handling and movement injuries (Total)**	**95**	**4,674**	**11.33**	**5.22**	**12.07**	**19.76**
Departments where patient handling injuries occur						
Inpatient adult wards	82	1,737	4.21	1.22	3.36	6.45
Inpatient adult critical care units	60	448	1.09	0.00	0.52	1.48
Outpatient acute care, emergency departments, urgent care	75	422	1.02	0.00	0.73	2.28
**Activities causing the most patient handling injuries**						
Positioning/repositioning in bed or stretcher	47	325	0.79	0.00	0.00	0.81
Transferring/lifting to/from bed or chair	45	290	0.70	0.00	0.00	0.78
Other	52	285	0.69	0.00	0.06	0.78
Lateral transfer of patient to/from bed	32	110	0.27	0.00	0.00	0.17
**Use of lifting equipment among injured employees**						
Unspecified	84	1,780	4.31	0.84	3.74	6.66
Using no equipment	89	2,387	5.79	2.13	6.05	9.62
Using equipment	71	507	1.23	0.00	0.91	2.04
**Severity of patient handling injuries**						
OSHA-recordable, unspecified	73	3,711	8.99	0.00	10.57	19.51
OSHA-recordable, days away from work	16	205	0.50	0.00	0.00	0.00
OSHA-recordable, job transfer/restriction	18	550	1.33	0.00	0.00	0.00
OSHA-recordable, all other cases	21	208	0.50	0.00	0.00	0.00
**Slips, trips, and falls injuries (Total)**	**99**	**3,972**	**9.63**	**5.57**	**8.21**	**14.35**
**Departments where slips, trips, and falls injuries occur**						
Inpatient adult wards	71	613	1.49	0.00	1.04	2.23
Non-patient care, maintenance	66	505	1.22	0.00	0.48	1.30
Inpatient operating rooms	61	382	0.93	0.00	0.55	1.45
**Sources causing the most slips, trips, and falls injuries**						
Hazard not recorded or not specified	79	663	1.61	0.21	1.53	3.42
Floor contaminant	70	558	1.35	0.00	0.89	1.80
Contact with object	60	281	0.68	0.00	0.42	0.95
Steps, stairs, or handrail	39	196	0.47	0.00	0.00	0.25
**Severity of slips, trips, and falls injuries**						
OSHA-recordable, unspecified	73	3016	7.31	0.00	6.59	13.96
OSHA-recordable, days away from work	22	210	0.51	0.00	0.00	0.00
OSHA-recordable, job transfer/restriction	19	489	1.19	0.00	0.00	0.00
OSHA-recordable, all other cases	24	257	0.62	0.00	0.00	0.00
**Workplace violence injuries (Total)**	**85**	**2,034**	**4.93**	**1.18**	**3.32**	**6.81**
**Departments where workplace violence injuries occur**						
Inpatient adult wards	64	635	1.54	0.00	0.53	1.92
Outpatient acute care, emergency departments, urgent care	58	372	0.90	0.00	0.21	1.53
Inpatient adult critical care units	41	154	0.37	0.00	0.00	0.42
**Common contributing factors among workplace violence injuries**						
Patient – contributing factor not specified	38	102	0.25	0.00	0.00	0.24
Patient – mental or behavioral health problems	16	60	0.15	0.00	0.00	0.00
Patient-cognitive dysfunction	18	31	0.08	0.00	0.00	0.00
Patient-other**	14	29	0.07	0.00	0.00	0.00
**Severity of workplace violence injuries**						
OSHA-recordable, unspecified	61	1,726	4.18	0.00	2.27	6.27
OSHA-recordable, days away from work	19	62	0.15	0.00	0.00	0.00
OSHA-recordable, job transfer/restriction	18	102	0.25	0.00	0.00	0.00
OSHA-recordable, all other cases	20	144	0.35	0.00	0.00	0.00

**Abbreviations:** OSHA = Occupational Safety and Health Administration.

* Injury incidence rate = (number of injuries/total facility full-time employees) × 10,000.

† OSHA-recordable injuries are defined as work-related injuries and illnesses that result in death, loss of consciousness, days away from work, restricted work activity or job transfer, medical treatment beyond first aid, or any substantial work related injury or illness that is diagnosed by a physician or other licensed health care professional.

§ Average worker-months = 125,041; worker-months are the number of full-time equivalent workers at a facility (or group of facilities) multiplied by the number of months worked within the reporting period. For example, a facility with 1,000 full-time equivalent workers has 12,000 worker-months in a 12 month reporting period.

¶ Pooled mean is the total number of incidents occurring at the facilities of interest within a given reporting period divided by the sum of the denominators for the same facilities over the same reporting period. A facility’s denominator is the product of a facility’s size (number of workers) and length of the facility’s participation (in months) within the given reporting period.

** Patient-other = the workplace violence incident involved a patient, and the contributing factor to the incident was mentioned in the report, but it did not fit into one of OHSN’s contributing factor categories.

## Footnotes

* OSHA-recordable injuries are defined as work-related injuries and illnesses that result in at least one of the following: death, loss of consciousness, days away from work, restricted work activity or job transfer, medical treatment beyond first aid, or a diagnosis by a physician or other licensed health care professional.

† Worker-months are defined as the number of full-time equivalent workers at a facility (or group of facilities) multiplied by the number of months worked within the reporting period. For example, a facility with a stable workforce of 1,000 full-time workers has 12,000 worker-months in a 12 month reporting period. If this same facility reported data for only 8 months, then they would have 8,000 worker-months. The total number of facility full-time employees is derived from the annual American Hospital Association survey and confirmed or modified by participating facilities to OHSN.

§ A pooled mean is the total number of incidents occurring at all the facilities of interest within a given reporting period divided by the sum of the denominators for the same facilities over the same reporting period. A facility’s denominator is the product of a facility’s size (number of workers) and length of the facility’s participation (in months) within the given reporting period.
